# Design Strategies of Hydrogen Evolution Reaction Nano Electrocatalysts for High Current Density Water Splitting

**DOI:** 10.3390/nano14141172

**Published:** 2024-07-09

**Authors:** Bao Zang, Xianya Liu, Chen Gu, Jianmei Chen, Longlu Wang, Weihao Zheng

**Affiliations:** 1 College of Electronic and Optical Engineering & College of Flexible Electronics (Future Technology), Nanjing University of Posts and Telecommunications, Nanjing 210023, China; 1223025003@njupt.edu.cn (B.Z.); b22030102@njupt.edu.cn (X.L.); b22020322@njupt.edu.cn (C.G.); chenjianmei@njupt.edu.cn (J.C.); 2 College of Advanced Interdisciplinary Studies & Hunan Provincial Key Laboratory of Novel Nano Optoelectronic Information Materials and Devices, National University of Defense Technology, Changsha 410073, China

**Keywords:** high current density, hydrogen evolution reaction, electrocatalyst, water splitting

## Abstract

Hydrogen is now recognized as the primary alternative to fossil fuels due to its renewable, safe, high-energy density and environmentally friendly properties. Efficient hydrogen production through water splitting has laid the foundation for sustainable energy technologies. However, when hydrogen production is scaled up to industrial levels, operating at high current densities introduces unique challenges. It is necessary to design advanced electrocatalysts for hydrogen evolution reactions (HERs) under high current densities. This review will briefly introduce the challenges posed by high current densities on electrocatalysts, including catalytic activity, mass diffusion, and catalyst stability. In an attempt to address these issues, various electrocatalyst design strategies are summarized in detail. In the end, our insights into future challenges for efficient large-scale industrial hydrogen production from water splitting are presented. This review is expected to guide the rational design of efficient high-current density water electrolysis electrocatalysts and promote the research progress of sustainable energy.

## 1. Introduction

Hydrogen, with its renewable, safe, high-energy density, and environmentally friendly properties, has emerged as the top alternative to traditional fossil fuel-based environments [1,2]. In contrast to other methods, such as steam methane reforming, biomass, and coal gasification for hydrogen production, water splitting for hydrogen production stands out as the most extensively adopted and developed method, especially known for its straightforward and non-polluting process [3,4,5]. However, the majority of HER electrocatalysts are evaluated at low current densities of 10 to 100 mA cm^−2^, which is far from satisfactory for industrial applications [6,7]. Particularly, it is remarkable to observe the significant benefits of extending it to industrial conditions for efficient water splitting at high current densities [8,9,10].

Initially, the HER is an electrochemical process driven by electron transfer through currents [11,12,13]. The efficiency of the electrolysis process increases with higher current densities, which reduces energy consumption [14,15,16,17]. Moreover, elevated current densities enhance hydrogen production efficiency, substantially boosting the volume of hydrogen produced per unit of time. As a general rule, industrial-scale applications require current densities above 500 mA cm^−2^, and high currents need to be combined with the lowest possible voltage [18,19]. For example, the U.S. Department of Energy plans to achieve 1600 mA cm^−2^ of industrial water electrolysis at 1.66 V by 2040.

Nonetheless, today’s industrial applications for hydrogen production by water splitting are confronted with numerous key challenges [20,21,22]. Firstly, commercially used catalysts still primarily consist of noble metals such as Pt, Ir, and Ru, noted for their high cost and limited availability [23]. Thus, it is crucial to advance catalysts made from non-noble metals that are plentiful on Earth. Secondly, the performance of non-precious metal catalysts still fails to meet the standards of precious metal catalysts because of a lack of sufficient activity [24,25,26]. Finally, catalysts are subject to serious stability issues under high current densities at the ampere level, particularly with the problem of catalyst shedding.

**Figure 1:**
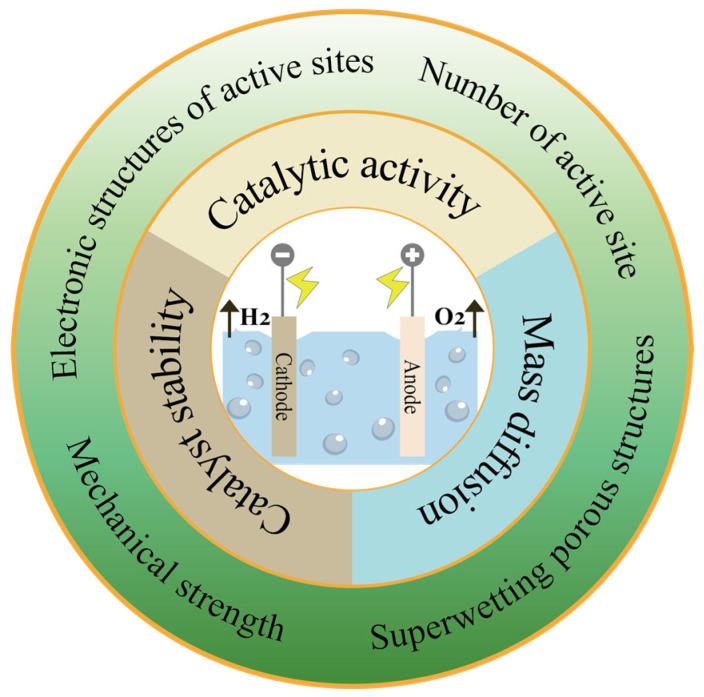
Challenges faced by electrocatalysts for HERs under industrial high current density water electrolysis.

Hence, the construction of non-precious metal-based electrocatalytic electrodes with enduring stability at ampere-level current densities is vital for water-splitting hydrogen production technologies aimed at industrial applications. This review summarizes several electrocatalyst design strategies to address the issues of catalytic activity, mass diffusion, and catalyst stability faced by non-precious metal-based electrocatalysts under industrial-scale high current densities (Figure 1).

## 2. Challenges of Electrocatalysts Faced for HERs under High Current Densities

The HER during water splitting is composed of several key steps, including the adsorption of reactants at the active sites, electron transfer from the support to the catalyst, and detachment of gas bubbles [27,28,29]. Moreover, high current densities can lead to structural and compositional changes, thereby decreasing the stability of electrocatalysts. To improve the performance of electrocatalysts under these conditions, it is essential to thoroughly analyze these critical challenges.

From the perspective of the electrochemical process, electrical conductivity is a fundamental factor influencing catalyst performance [30,31]. Under high current density conditions, multi-electron electrochemical reactions occur intensely and rapidly, leading to quick occupation of active sites on the catalyst’s surface and facilitating swift charge consumption [32,33]. If electron transfer is insufficiently rapid, charge consumption will surpass charge supply, thereby impacting the efficiency of the catalytic reaction. The charge transfer rate is determined by the conductivity and interfacial resistance at the support-catalyst interface. Conductivity is influenced by various factors. Firstly, the crystal structure at the support–catalyst interface plays a role in electron transfer and conduction. For instance, defects or impurities within the crystal lattice can create additional energy levels, impacting electron conductivity [34]. Secondly, the surface morphology affects electron transport at the support–catalyst interface [35]. Nanostructures with a high specific surface area and numerous active sites can offer more electron transport pathways, thereby enhancing conductivity [36]. Lastly, a significant difference in Fermi energy levels between the conductive support and the semiconductor catalyst can lead to the formation of a Schottky barrier at the interface, preventing efficient electron transfer from electron-rich to electron-deficient regions and impeding charge transfer at the interface [37]. As a result, an additional overpotential is required to overcome the energy barrier, which deteriorates the electrocatalytic performance at high current densities.

According to Faraday’s law, the rate of hydrogen production is directly proportional to the current density [38,39]. Therefore, for HERs at high current densities, the bubble detachment performance has always been an important factor to consider. Similar to electron transfer, when bubble detachment is hindered, the blocked bubbles will limit the catalytic performance of the electrocatalyst [40,41]. In particular, gas bubbles cling to the electrode surface and gather at the interface between the electrocatalyst and the electrolyte, significantly hindering liquid mass transfer and extensively obstructing the active sites on the electrocatalyst. The presence of these bubbles on the electrode surface reduces the effective surface area of the electrocatalyst available for the reaction, thereby lowering the efficiency of the electrocatalytic process [42,43,44]. Moreover, this large-scale coverage undoubtedly leads to poor contact between the electrolyte and the electrode and increases the internal resistance of the system [45,46].

When conducting water splitting for hydrogen production at high current densities, electrocatalysts face stability challenges categorized into chemical and mechanical stability. In terms of chemical stability, high current density conditions can alter the crystal structure, surface morphology, and pore architecture of the catalyst, consequently reducing its stability [47,48,49]. Moreover, the substantial electron transfer occurring under high current densities accelerates the catalyst’s reconstruction process, imposing more severe effects on the catalyst. Regarding mechanical stability, the significant impact of electrochemical shear forces, along with the thermal effects encountered at high current densities, may cause insufficient interfacial adhesion between the catalyst and the support, making the catalyst prone to detachment during the reaction. Furthermore, the heat generated during the reaction raises the electrode’s temperature, which not only compromises the catalyst’s chemical stability but may also change the material’s physical properties, like causing swelling or softening, further weakening the bond between the catalyst and the support [50,51].

## 3. Design Strategies of Electrocatalysts for HERs under High Current Densities

The above-mentioned series of problems cause the hydrogen production performance of electrocatalysts at a high current density to be far from ideal. Consequently, to enhance hydrogen production efficiency under high current densities, additional advancements in catalyst design are required. Catalytic activity, mass diffusion, and catalyst stability represent the three principal challenges to achieving water splitting at high current densities. To surmount these obstacles, it is imperative to judiciously design water-splitting electrocatalysts that simultaneously enhance activity and stability. Consequently, this section delves into electrocatalyst design strategies in terms of the electronic structure of active sites, the number of active sites, superwetting structures, and mechanical strength. These strategies are critical for optimizing the catalysts’ performance by addressing the specific challenges posed at high current densities.

### 3.1. Tuning Electronic Structure and Crystal Phase of the Catalyst for Enhancing Intrinsic Activity

**Figure 2:**
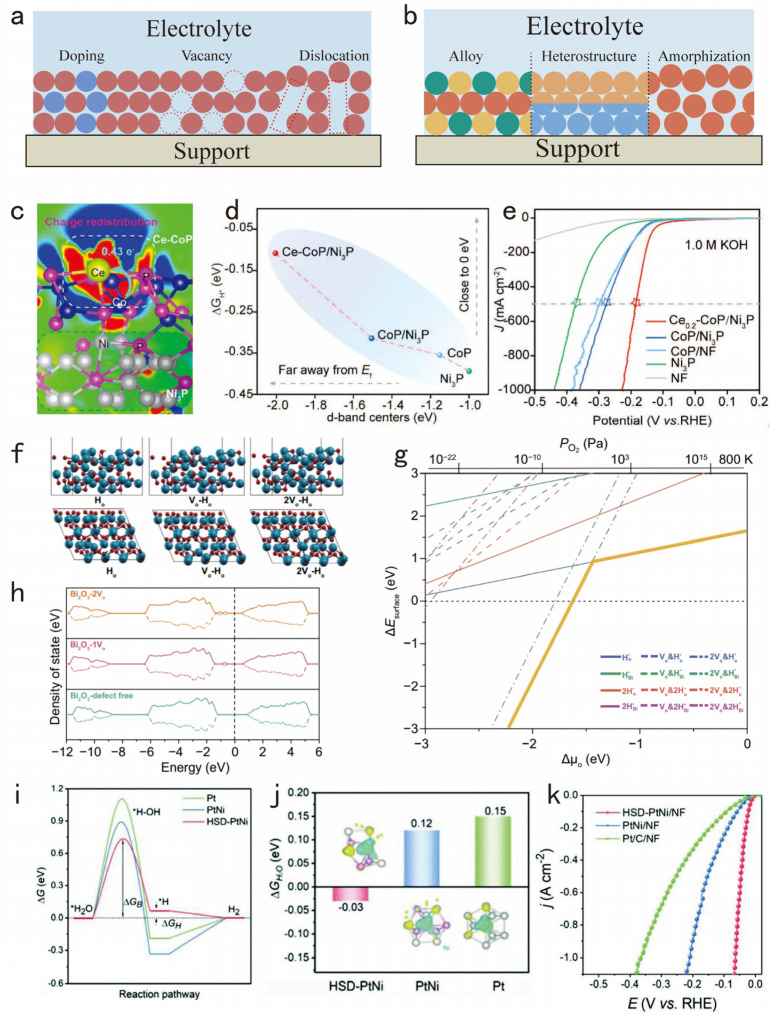
Strategies for dealing with rapid electron transfer at high current densities. (**a**) Defect engineering (including heteroatom doping, vacancy engineering, and dislocation modulation). (**b**) Alloying, heterostructure, and amorphization. (**c**) Differential charge density 2D slice of the CeCoP/Ni_3_P. (**d**) The tailoring relationship between ∆G_H*_ and d-band centers (ε_d_). (**e**) J–V curves of Ce_0.2_-CoP/Ni_3_P@NF, CoP/Ni_3_P@NF, CoP/NF, Ni_3_P@NF, and bare NF. Adapted with permission from [61], Copyright 2022 Wiley-VCH GmbH, Hoboken, NJ, USA. (**f**) Models of hydrogen atom adsorption on Bi_2_O_3_ (010) surfaces in both defect-free and oxygen vacancy-incorporated states. (**g**) The calculated relative surface energies as functions of the chemical potential of oxygen (∆μo). (**h**) The spin-polarized electronic density of states for Bi_2_O_3_ surfaces with varying oxygen vacancy coverage. Adapted with permission from [64], Copyright 2022 Springer Nature, Berlin, Germany. (**i**) Gibbs free energy diagram for HER on the (111) plane of HSD-PtNi, PtNi, and Pt. (**j**) Comparisons of the derived adsorption Gibbs free energies of H_2_O on HSD-PtNi, PtNi, and Pt. (**k**) HER linear sweep voltammetry (LSV) curves with iR correction. Adapted with permission from [65], Copyright 2022 Wiley-VCH GmbH, Hoboken, NJ, USA.

For high-performance HER electrocatalysts operating at high current densities, possessing high intrinsic activity and excellent electrical conductivity are key to their performance [52]. Achieving these characteristics requires the precise modulation of the electronic structure, which not only significantly enhances the intrinsic activity of the catalysts but also improves its conductivity [53,54], thus markedly boosting the effectiveness and durability of the water electrolysis process under high current density scenarios [55]. Strategies for optimizing the electronic structure and crystal phase of the catalyst are diverse, including but not limited to defect engineering, alloying, heterostructure, and amorphization (Figure 2a,b).

By meticulously designing defects in the electrocatalysts, such as through heteroatom doping, vacancy engineering, and dislocation modulation, the electronic structure can be effectively tuned, and the adsorption energy of reaction intermediates can be optimized [56]. Heteroatom doping strategies entail incorporating non-intrinsic heteroatoms into the catalyst’s lattice. Specifically, this approach can modify the material’s electronic environment by either replacing original lattice positions or embedding into lattice interstices [57].

Noble metal atoms, such as Pt and Ru, are extensively used in doping strategies due to their superior catalytic properties [58]. Nevertheless, the high expense of these noble metals restricts their viability for large-scale applications [59]. Consequently, there is increasing interest within the scientific community in finding more cost-effective alternatives among transition metals, which have also shown exceptional abilities in modulating electronic structures and enhancing electron transfer [60]. For instance, Zhang et al. effectively synthesized a cerium-doped CoP/Ni_3_P composite through a combination of corrosion, electrodeposition, and phosphorization calcination methods [61]. Through Ce doping, the electronic structure of the CoP/Ni_3_P electrocatalyst was modulated to improve the electron transfer process (Figure 2c). This Ce doping efficiently redistributed charges and adjusted the d-band center, accelerating the H_2_O dissociation step and enhancing the kinetics of alkaline water splitting at high current densities (Figure 2d). Furthermore, this adjustment of the electronic structure resulted in an overpotential of just 225 mV at a high current density of 1000 mA cm^−2^ (Figure 2e), enabling a stable operation in alkaline electrolytes for 200 h, thus demonstrating outstanding catalytic activity and stability. Additionally, doping with non-metal elements is an effective strategy to improve the performance of electrocatalysts [62].

In addition to heteroatom doping, the introduction of vacancies has also been broadly utilized to modify the electronic structure of electrocatalysts [63]. Interestingly, the relationship between different concentrations of vacancies and HER activity is inherently linked. For example, Sun and his team used Bi_2_O_3_ nanosheets as a model system to investigate the effect of different concentrations of oxygen vacancies (V_o_) created by plasma irradiation on HER performance [64]. Initially, the introduction of V_o_ was observed to enhance charge transfer and increase the number of active sites for hydrogen adsorption, thereby boosting HER activity. This improvement is due to alterations in the catalyst’s electronic structure and surface chemistry caused by the presence of V_o_ (Figure 2f–h). However, the research also identified a critical threshold for V_o_ concentrations. Beyond this saturation point, further increases in V_o_ concentrations resulted in a marked decrease in HER activity, which maximizes HER performance by balancing the advantages of increased active sites and improved charge transfer with the negative impacts of excessive V_o_.

Moreover, in the realm of defect engineering for electrocatalysis, the strategic incorporation of dislocation networks emerges as a critical innovation for enhancing the efficacy of HERs. Yang’s research emphasized that an innovative fabrication method, employing millisecond laser direct-write synthesis in a liquid nitrogen setting, was used to create PtNi alloy nanoparticles with complex dislocation networks on nickel foam substrates [65]. This advanced design of dislocation networks serves two primary functions. First, it significantly enhances the fundamental process of alkaline HERs by introducing substantial tensile-compressive coupling strains. From a kinetic perspective, HSD-PtNi exhibits a much lower water dissociation barrier (Δ_GB_, 0.73 eV) compared to PtNi and Pt (Figure 2i). Second, the electron density of Ni atoms under maximum tensile strain is considerably decreased, leading to stronger electronic interactions between the H_2_O molecules and the Ni sites (Figure 2j). Additionally, it effectively stabilizes the surface dislocations against the stresses of high current densities, indicating that HSD-PtNi exhibits much lower overpotentials at high current densities compared to PtNi/NF and Pt/C/NF, needing only a minimal overpotential of 63 mV to reach an ultra-high current density of 1 A cm^−2^ (Figure 2k).

In addition to defect engineering, strategies such as alloying, heterostructure, and amorphization have also shone in the direction of facilitating charge transfer and are considered to be powerful tools for the electronic structure modulation of electrocatalysts, laying a solid foundation for the achievement of unparalleled high current density hydrogen evolution efficiencies [66].

**Figure 3:**
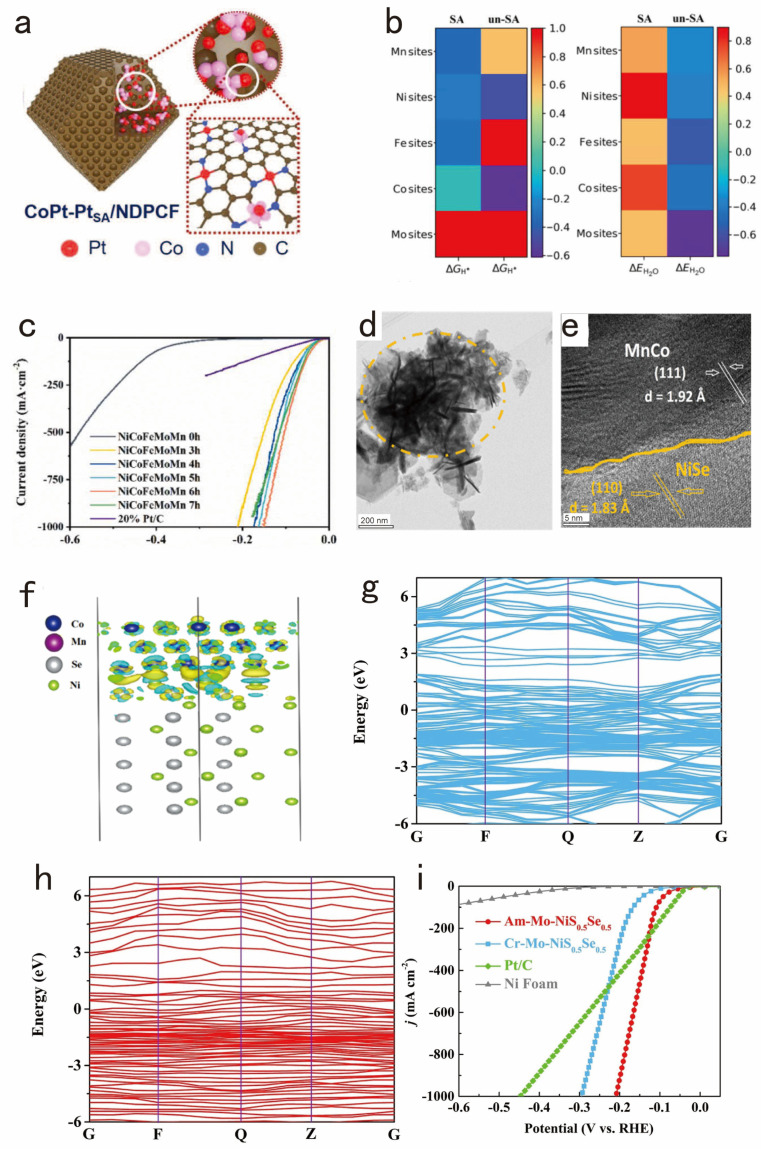
(**a**) The bimetallic CoPt-alloy nanocrystals and PtSA were confined inside the NDPCF. Adapted with permission from [67], Copyright 2022 Wiley-VCH GmbH, Hoboken, NJ, USA. (**b**) The colored ΔG_H*_ and ΔE_H2O_ comparisons of all possible active sites on regions of elemental segregation and other regions of high-entropy alloy models; the pure red area means this site is not easy to adsorb. (**c**) HER polarization curves for nanoporous NiCoFeMoMn and commercially available Pt/C in 1 M KOH solution. Adapted with permission from [68], Copyright 2022 Elsevier, Amsterdam, The Netherlands. (**d**) TEM and (**e**) HRTEM images of MnCo/NiSe electrode. (**f**) Electron density redistribution of the interface made by MnCo and NiSe. Adapted with permission from [70], Copyright 2022 Elsevier, Amsterdam, The Netherlands. The band structure of the (**g**) Cr-Mo-NiS_0.5_Se_0.5_ and (**h**) Am-Mo-NiS_0.5_Se_0.5_. (**i**) HER polarization curves. Adapted with permission from [71], Copyright 2022 Wiley-VCH GmbH, Hoboken, NJ, USA.

Alloying is considered to be an efficient way to modulate electronic structures and enhance electrical conductivity, offering a cost-effective solution by alloying noble metals with transition metals. This synergistic effect not only alleviates the high cost of precious metal catalysts but also facilitates electron transfer, tunes the Fermi energy levels, and enhances the intrinsic activity of the catalysts. Li et al. synthesized CoPt-PtSA/NDPCF by integrating Pt nanocrystals into the ZIF-67 precursor, followed by high-temperature calcination [67]. This fabrication approach ensured uniformity and optimal dispersion, thereby enhancing the efficiency of reactive active sites (Figure 3a). Moreover, surface active sites were optimized through the precise control of Pt and Co ratios and nanostructures. CoPt-PtSA/NDPCF demonstrated reduced overpotentials and higher current densities compared to commercially available 10% Pt/C catalysts, indicating a superior hydrogen production performance in both alkaline and acidic environments. With the continuous advancement in alloy research, several metal alloys have emerged and evolved over time. High-entropy alloys (HEAs) have been introduced as a novel class of alloy materials. HEAs, composed of five or more metal elements mixed, exhibit unique chemical and physical properties due to their solid-solution phases. Zhao et al. developed a nanoporous NiCoFeMoMn high-entropy alloy through a one-step dealloying process [68]. Additionally, they constructed nanoporous high-entropy alloy (np-HEA) models for both surface-active (SA) and non-surface-active (un-SA) configurations. The Gibbs free energies of hydrogen adsorption were computed for all feasible active sites on the material’s surface, depicted in Figure 3b. It is evident that an SA in np-HEA plays a pivotal role in the hydrogen adsorption characteristics. The alloy exhibited outstanding HER properties in electrolytic water tests, achieving a current density of 1000 mA cm^−2^ at an overpotential of just 150 mV in a 1 M KOH solution (Figure 3c) and demonstrating a low Tafel slope of 29 mV dec^−1^.

Heterostructures further brighten the way to optimize electronic structures and facilitate charge transfer. By cleverly combining different materials, the heterostructures provide the fundamental basis for improved water dissociation and hydrogen adsorption, which greatly enhances the catalytic efficiency under high current density conditions. For instance, Zhou et al. utilized porous interlaced Co_2_N nanosheets and Fe_2_P nanoparticles to fabricate an array of Fe_2_P/Co_2_N heterostructures [69]. The surface of these heterostructures is rich in Fe sites, and DFT calculations indicate that the interfacial interaction between Fe_2_P and Co_2_N enhances the hydrogen binding energy (∆G_H*_) on the Fe sites, thereby improving the catalyst’s HER performance. In another study, Li’s team electrodeposited a MnCo layer onto NiSe samples [70], which effectively roughens the NiSe surface (Figure 3d,e). Interestingly, the MnCo deposition on NiSe leads to a reduction in the electron density surrounding the Ni and Se atoms. This implies electron transfer between materials of varying electronegativity, creating a heterogeneous structure that enhances the catalyst’s HER efficiency. Furthermore, the Nyquist plots indicate a marked decrease in Rct values for MnCo/NiSe (Figure 3f), indicating higher electrical conductivity compared to NiSe, thereby facilitating enhanced charge transfer.

Amorphization introduces disorder in the crystal matrix, which reveals a large number of active sites and alters the electronic properties in favor of HERs. This disorder strengthens the adsorption energy regulation and accessibility of the reaction products, greatly increasing the intrinsic activity of the catalysts. For instance, Hu’s team prepared amorphous Mo-doped NiS_0.5_Se_0.5_ nanosheets (Am-Mo-NiS_0.5_Se_0.5_) and uniformly wrapped them on nanorods [71]. The creation of Am-Mo-NiS_0.5_Se_0.5_ composites increased active sites and altered the local electronic structure, thereby enhancing intrinsic activity. X-ray photoelectron spectroscopy (XPS) patterns visually showed reduced electron density around Mo atoms and accumulation around Ni atoms in the amorphous structure, indicating modified electron distribution affecting electronic structure. Meanwhile, DFT theoretical calculations also show that Am-Mo-NiS_0.5_Se_0.5_ has no obvious indirect band gap, which confers its excellent performance (Figure 3g,h). It only needs overpotentials of 209 for HERs at 1000 mA cm^−2^ (Figure 3i), which demonstrates a hydrogen production performance that is superior to that of crystalline structures.

### 3.2. Designing the Interface of the Electrocatalysts for Exposing a Large Number of Active Sites

**Figure 4:**
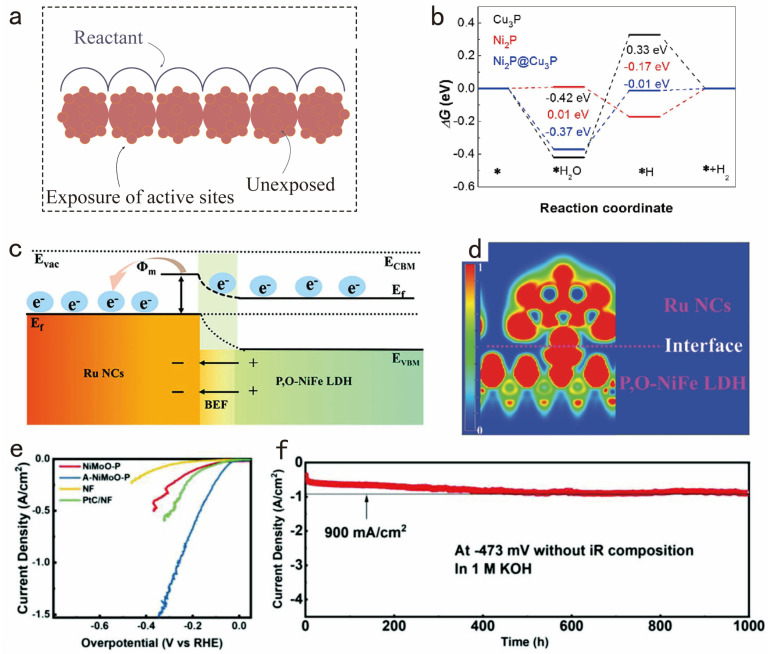
(**a**) Schematics showing exposure of active sites. (**b**) The free energy diagrams of H_2_O and H adsorbing. Adapted with permission from [72], Copyright 2023 Elsevier, Amsterdam, The Netherlands. (**c**) Schematic drawing of electron redistribution in Ru NCs/P,O-NiFe LDH/NF. (**d**) Differential charge density of Ru NCs/P,O-NiFe LDH/NF, with iso-values of 0 and 1 indicating low and high electron localization, respectively. Adapted with permission from [73], Copyright 2023 Wiley-VCH GmbH, Hoboken, NJ, USA. (**e**) LSV curves of A-NiMoO-P and other catalysts. (**f**) Chronopotentiometric *i-t* curve of A-NiMoO-P at the constant potential of −473 mV. Adapted with permission from [74], Copyright 2024 Wiley-VCH GmbH, Hoboken, NJ, USA.

Since the overall activity of an electrocatalyst also depends on the number of its active sites, the interface of the electrocatalyst should be carefully designed to expose a large number of active sites (Figure 4a). By optimizing the interfaces between the materials in the electrocatalyst and other materials, the electron transport efficiency can be improved. For example, Wang et al. developed a non-precious Ni_2_P@Cu_3_P heterostructure constructed by in-situ phase conversion for electrochemical HERs [72]. It is noteworthy that the 3D nanowire array architecture offers a substantial specific active surface area conducive to rapid charge and mass transfer, thereby enhancing the kinetics of the HER process. Experimental data demonstrated that Ni_2_P@Cu_3_P exhibited enhanced HER performance relative to pure Ni_2_P and Cu_3_P (Figure 4b). Moreover, DFT simulations indicated that Ni_2_P@Cu_3_P exhibits more favorable free energies for facilitating alkaline HERs, thereby validating its effective modification at the active site (Figure 4c). In addition, Zang et al. considered the possibility of promoting unidirectional electron transfer by means of a built-in electric field to ensure electron enrichment [73]. The presence of a heterojunction between Ru nanoclusters and P,O-NiFe LDH/NF (as shown in Figure 4d) has been verified, serving as a catalyst for enhanced electron transfer during electrocatalysis.

Furthermore, in addition to the active sites inherent in the catalysts described above, an active site in situ refreshing strategy can also provide multistage active sites. Shi et al. significantly enhanced the performance and stability of the electrocatalyst by continuously refreshing the active sites during the electrochemical reaction [74]. Specifically, the exposure of the embedded active sites can be promoted by the dynamic balance of dissolution and deposition in the electrochemical reaction, which effectively avoids the coverage and deactivation of the active sites. During the activation process of A-NiMoO-P, part of Ni(PO_3_)_2_ is converted to Ni_2_P under electrochemical conditions, which continuously exposes fresh active sites and ensures the catalyst has an extremely high current density of 1500 mA cm^−2^ at a low overpotential of 340 mV (Figure 4e). It is surprising to note that the catalyst also has an ultra-long durability of hydrogen evolution for at least 1000 h, as shown in Figure 4f.

### 3.3. Designing Superwetting Porous Structure for Accelerating Bubble Detachment

**Figure 5:**
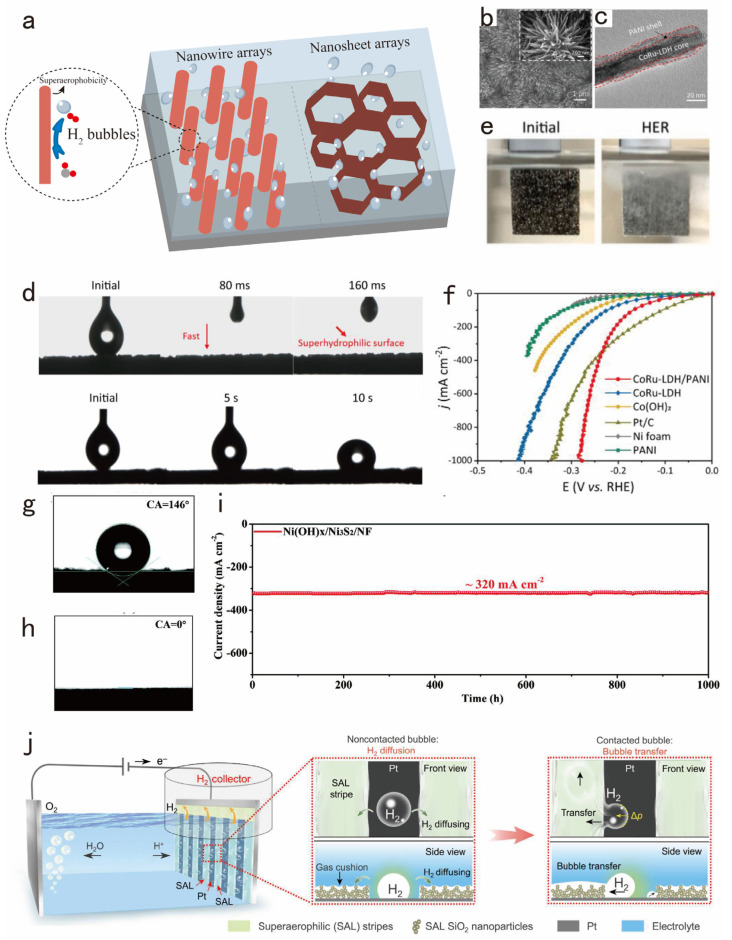
(**a**) Physical models of morphology engineering, such as nanowire arrays and nanosheet arrays, to solve the problem of bubble detachment under a high current density. (**b**) SEM and (**c**) TEM images of CoRu-LDH/PANI. (**d**) Contact angle measurements of CoRu-LDH/PANI and Pt/C. (**e**) The release of H2 bubbles on CoRu-LDH/PANI. (**f**) Polarization curves based on geometric area in 1 M KOH. Adapted with permission from [77], Copyright 2022 Elsevier, Amsterdam, The Netherlands. (**g**,**h**) Contact angles of Ni_3_S_2_ and Ni(OH)_x_/Ni_3_S_2_. (**i**) Chronopotentiometry curve of the Ni(OH)x/Ni_3_S_2_/NF recorded at ≈320 mA cm^−2^ for 1000 h. Adapted with permission from [78], Copyright 2022 Wiley-VCH GmbH, Hoboken, NJ, USA. (**j**) Schematic of the SAL/flat Pt electrode with enhanced mass transfer. Adapted with permission from [79], Copyright 2023 The American Association for the Advancement of Science, Washington, DC, USA.

At high current densities, abundant bubbles that are unable to detach will undoubtedly block the exposure of catalyst active sites, which seriously affects the mass transfer of the system [75]. An important issue in electrocatalysis is enhancing the efficient release of bubbles from the catalyst surface. This review investigates this challenge through the perspective of the catalyst structure, particularly emphasizing the nanoarray configuration and porous architecture as crucial aspects (Figure 5a).

Initially, nanowire arrays possess distinct surface properties, such as a large surface area and high aspect ratio, which can profoundly influence the electrolyte’s flow dynamics and expedite its penetration. For instance, Yin et al. destroyed the smooth morphology of the nanoneedle structure by calcination at high temperatures [76], which strengthened the surface wettability of the catalyst and effectively promoted the release of gas bubbles. Liu’s group reported a polyaniline (PANI)-coated CoRu-LDH (CoRu-LDH/PANI) nanowire array electrocatalyst [77]. The nanowire array structure significantly reduces the attachment time of gas bubbles to the catalyst surface due to its highly oriented surface architecture. Upon chemically polymerizing coarse PANI onto the nanowires (Figure 5b), there was a remarkable enhancement in the catalyst’s capability to facilitate bubble detachment. Coarse PANI introduced micro- and nano-scale roughness to CoRu-LDH (Figure 5c), thereby increasing the electrocatalyst’s surface area and providing additional sites for water molecule adsorption to facilitate water film formation. Furthermore, the amine-rich PANI reinforced the interface between CoRu-LDH and PANI, which promotes rapid electrolyte transport and efficient gas bubble desorption, as illustrated in Figure 5d. Moreover, only a minimal amount of bubble adhesion occurs on its surface (Figure 5e), which effectively prevents the clogging of active sites to some extent. In addition, the CoRu-LDH/PANI exhibits excellent hydrogen production performance, achieving a high current density of 1000 mA cm^−2^ with a low overpotential of only 275 mV (Figure 5f). Furthermore, nanosheet arrays represent some of the most advanced non-noble metal electrocatalysts, owing to their superhydrophilicity, microporous nature, and self-supporting structure. For example, Xin et al. developed a self-supported microporous Ni(OH)_X_/Ni_3_S_2_ heterostructure electrocatalyst using an electrochemical method [78]. As depicted in Figure 5g,h, the contact angle of Ni(OH)_X_/Ni_3_S_2_ is 0°, indicating its significant superhydrophilicity compared to the hydrophobic Ni_3_S_2_ electrode. Furthermore, the Ni(OH)_X_/Ni_3_S_2_/NF catalyst exhibited long-term stability exceeding 1000 h (Figure 5i).

Although the aforementioned electrocatalysts excel in promoting bubble detachment, the released bubbles often exhibit disorderly behavior, and few studies have addressed how to control the departure of these bubbles from the electrolyte. Addressing this challenge, Jiang’s team introduced a superaerophilic/superaerophobic (SAL/SAB) synergistic electrode configuration [79] (Figure 5j). The air cushion on the SAL stripes acts like a sky bridge, providing a fast path for the bubbles to leave the reaction system directly. When the bubbles contact the SAL stripes, they are transported to the external surface in a very short period of time, as if they were sitting in a car propelled by Laplace pressure.

**Figure 6:**
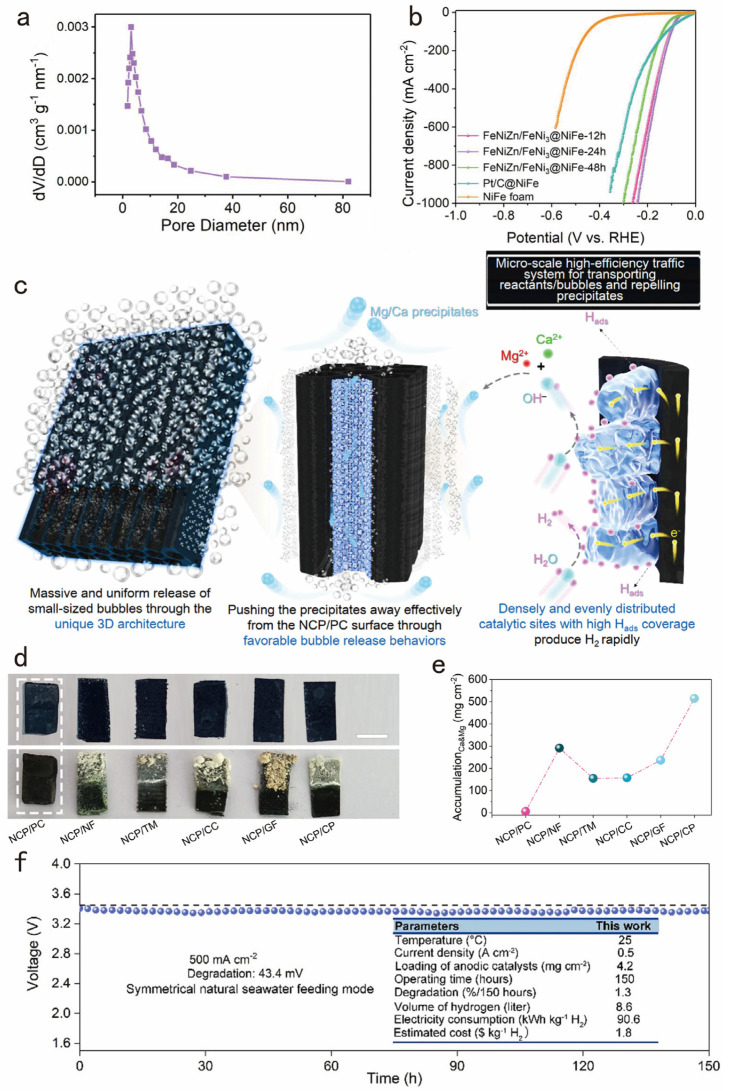
(**a**) BJH pore size distribution curve of the FeNiZn/FeNi_3_@NiFe sample. (**b**) LSV curves of FeNiZn/FeNi_3_@NiFe samples, Pt/C@NiFe, and NiFe foam in 1.0 M KOH solution at the scan rate of 5 mV s^−1^. Adapted with permission from [80], Copyright 2023 The Author(s). (**c**) Schematic diagrams of densely and evenly distributed catalytic sites with high H_ads_ coverage produce H_2_ rapidly. (**d**) Pictures of various cathodes after the 10 h of eNSR. Scale bar: 0.5 cm. (**e**) A_Ca&Mg_ values for various cathodes after the long-term eNSR testing. (**f**) Long-term stability of NCP/PC||DSA under a fixed j of 500 mA cm^−2^. Adapted with permission from [81], Copyright 2024 The Author(s).

Obviously, porous structures are more likely to promote bubble detachment. Yuan and colleagues developed a multidimensional FeNiZn alloy with nanoporous interpenetrating phases and a FeNi3 intermetallic heterostructure on NiFe foam to enhance water-splitting efficiency [80]. As shown in Figure 6a, it can be found that several second-order pores in the FeNiZn/FeNi_3_@NiFe sample. Furthermore, FeNiZn/FeNi_3_@NiFe exhibits remarkable bifunctional activities for water splitting, featuring exceptionally low overpotentials for HERs and robust durability over 400 h of testing in an alkaline solution (Figure 6b).

The smaller bubble sizes implied that the bubbles had a shorter residence time at the reaction interface, which improved the detachment efficiency of the bubbles. Tang et al. introduced an essential microscopic bubble/precipitate traffic system for robust anti-precipitation seawater reduction [81]. Due to its unique 3D H_2_-evolving architecture, NCP/PC provides significant benefits, enhancing electrocatalysis efficiency, facilitating H_2_ gas release, and offering superb anti-precipitation properties (Figure 6c). Noticeably, after prolonged electrolysis, the surface of NCP/PC remains clean, unlike the other five NCP-based cathodes, which are coated with dense Mg^2+^/Ca^2+^ precipitates (Figure 6d,e), demonstrating NCP/PC’s strong anti-precipitation capability. Moreover, this long-term seawater electrolysis durability surpasses that of the most advanced natural seawater electrolyzer, which operates at a current density of 500 mA cm^−2^ for 100 h (Figure 6f).

### 3.4. Modulating Structure of Electrocatalysts for Enhancing the Chemical/Mechanical Strength

**Figure 7:**
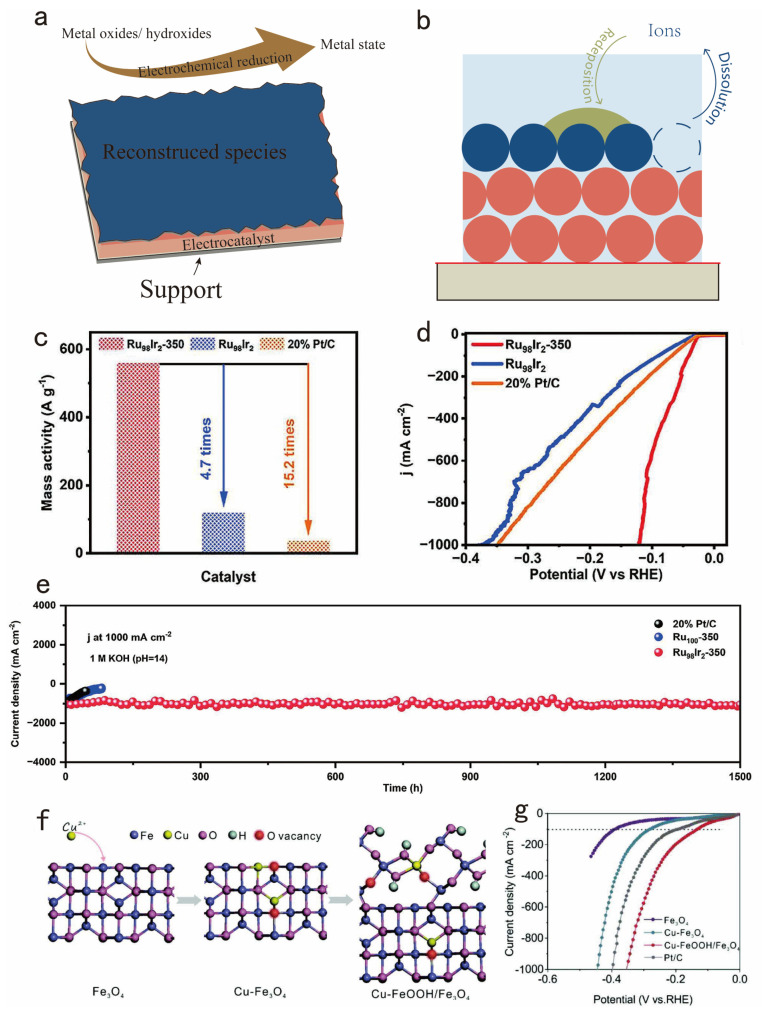
Schematics show key aspects for the enhancement of chemical and mechanical stability of electrocatalysts. Strategies such as (**a**) electrochemical reduction and (**b**) redeposition of dissolved materials and ionic modulation of the reconstruction to enhance the chemical stability. (**c**) Mass activity value at an overpotential of 100 mV. (**d**) LSV curves of the as-synthesized Ru_98_Ir_2_-350, Ru_98_Ir_2_ samples, and commercial Pt/C catalyst. (**e**) i-t continuous durability of Ru_98_Ir_2_-350, Ru_100_, and commercial Pt/C at 1000 mA cm^−2^. Adapted with permission from [87], Copyright 2023 The Authors. Advanced Science published by Wiley-VCH GmbH, Hoboken, NJ, USA. (**f**) Schematic diagram of the structural transformation at an atomic level. (**g**) The iR-corrected LSVs of the Cu-FeOOH/Fe_3_O_4_ catalyst in 1 M KOH. Adapted with permission from [88], Copyright 2022 The Authors. Advanced Energy Materials published by Wiley-VCH GmbH, Hoboken, NJ, USA.

It is well known that the surface reconstruction process of electrocatalysts is essentially a chemical reaction [82]. Therefore, it is particularly significant to observe the reconstruction process under electrochemical conditions. A great deal of research results show that the original catalyst experiences dynamic reconstruction and generates real active sites during the reaction process, which optimizes the adsorption, activation, and desorption behaviors during the catalytic process to a certain extent and thus boosts the HER performance of the electrocatalysts [83,84]. This type of electrocatalyst before reconstruction is called a “pre-catalyst”. Therefore, it is necessary to artificially intervene to adjust the reconstruction process of pre-catalysts to obtain more active sites [85]. This review summarizes effective modulation strategies to promote the surface reconstruction process to augment the HER activity. The modulation strategies can be classified into electrochemical activation, redeposition of dissolved materials, and ionic modulation of the reconstruction (Figure 7a,b).

In some situations, electrochemical activation can prompt the formation of new active phases on the catalyst surface, which may have superior HER properties to the original material [86]. Through appropriate electrochemical treatment, thin layers of metal hydrides or oxides can be formed on the catalyst surface, and these specific phases may be more active for HER reactions. For example, Lu et al. utilized the reducing capability of sodium borohydride (NaBH_4_) to simultaneously reduce Ru^3+^ and Ir^3+^ in metal salts, forming Ru_98_Ir_2_ in situ, which could subsequently be oxidized to produce Ir-doped, partially oxidized Ru metallic aerogels [87]. Compared to Pt/C, Ru_98_Ir_2_-350 exhibits a higher mass activity value, as shown in Figure 7c. Notably, the Ru_98_Ir_2_-350 sample delivered a superior catalytic activity at current densities of 1000 mA cm^−2^, only requiring overpotentials of 121 mV (Figure 7d). In addition, the Ru_98_Ir_2_-350 demonstrated excellent CV stability at 1000 mA cm^−2^, which is illustrated in Figure 7e.

It is well known that high oxidation/reduction potentials and corrosive electrolytes cause elements to dissolve and react with the electrolyte. The leached components are then redeposited onto the catalyst surface, leading to a dynamically reconstructed surface that offers high performance and stability [62]. For instance, Yang et al. induced abundant defects and unsaturated sites by incorporating an amorphous structure on the surface of a catalyst to enhance HER activity [88]. A schematic diagram illustrating the crystal structure transformation at an atomic level showed that the atomic radius of Cu is similar to that of Fe, facilitating the substitution of Fe atoms for Cu atoms. This indicates the leaching of Fe and Cu species during the formation of the CuFeOOH/Fe_3_O_4_ catalyst (Figure 7f). Notably, the Cu-FeOOH/Fe_3_O_4_ catalyst exhibits superior HER activity in 1 M KOH, achieving an ultra-low overpotential of 285 mV at a current density of −500 mA cm^−2^ (Figure 7g).

For high-current-density electrolytic hydrogen evolution, electrocatalysts are exposed to more corrosive electrolytes, higher reaction temperatures, and higher current densities, which inevitably induce the deactivation and detachment of catalytic substances from the electrocatalysts [89]. Therefore, the designed electrocatalysts should have excellent mechanical stability in addition to high activity. Self-supported electrocatalysts are outstanding in high current density due to their unique structural design [90]. The following discussion presents the research progress of self-supported electrocatalysts in terms of self-supported substrates and nanostructured catalysts.

**Figure 8:**
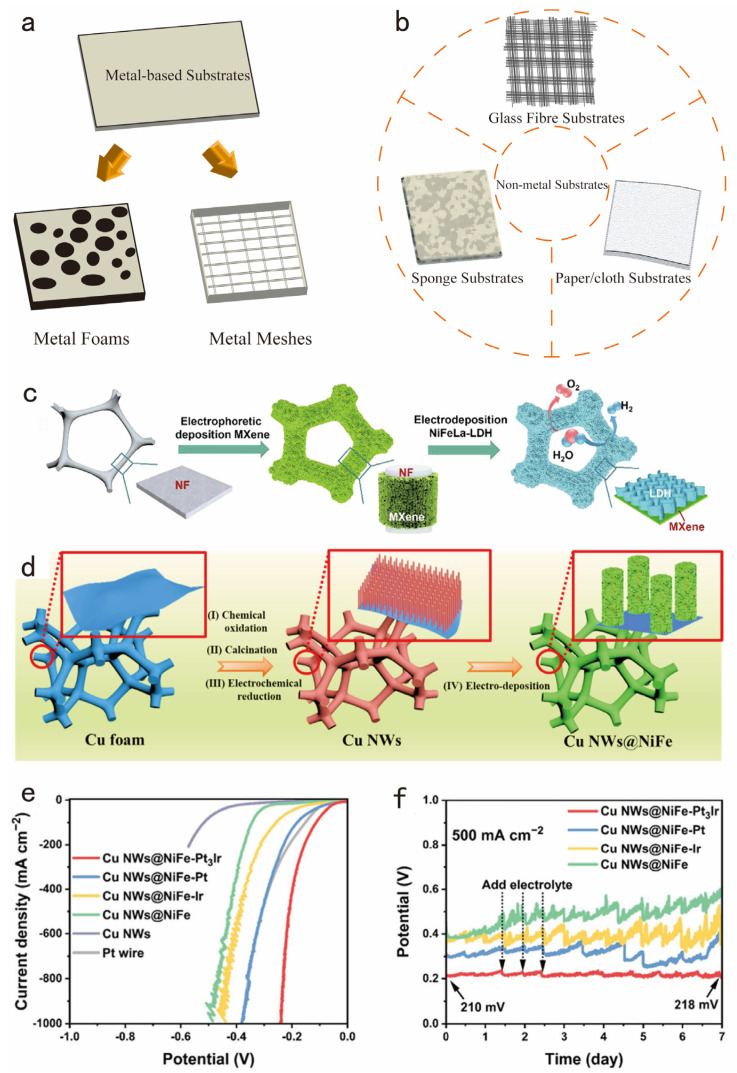
Schematics show self-supported substrates, including (**a**) metal-based substrates and (**b**) non-metal substrates. (**c**) The schematic illustration depicts the fabrication process of NiFeLa-LDH/v-MXene/NF. Adapted with permission from [93], Copyright 2022 Elsevier, Amsterdam, The Netherlands. (**d**) Schematic illustration for the synthetic process of Cu NWs@NiFe. (**e**) LSV curves of the Cu NWs@NiFe-Pt_3_Ir, Cu NWs@NiFe-Pt, Cu NWs@NiFe-Ir, Cu NWs@NiFe, Cu NWs, and Pt wire, respectively. (**f**) Chronopotentiometry curves of the Cu NWs@NiFe-Pt_3_Ir, Cu NWs@NiFe-Pt, Cu NWs@NiFe-Ir, and Cu NWs@NiFe. Adapted with permission from [94], Copyright 2022 Elsevier, Amsterdam, The Netherlands.

Selecting an appropriate substrate is crucial for the preparation of self-supported electrocatalysts. Metal-based catalysts, such as metal foams and metal meshes, have attracted widespread attention (Figure 8a). Besides nickel foam, other metal-based foams like iron foam (IF), cobalt foam (CF), and copper foam (CFM) have also been extensively studied.

Apart from the metallic foams mentioned above, other non-metallic substrates are being explored. For example, insulating materials such as flexible glass fibers, paper or cloth, and sponges (Figure 8b) as substrates are promising materials. Hao et al. realized the significance of flexible materials in recognizing the long-term stability of electrocatalysts [91]. With the help of precision instruments, they achieved the deposition of 3D dandelion gown-like Fe_1_-Ni_2_P onto a flexible glass fiber substrate. The electrocatalyst prepared not only possessed advantages such as corrosion resistance and good elasticity but also exhibited a loose and porous characteristic, which contributed to the enhancement of hydrogen evolution of the electrocatalyst at high current densities.

Furthermore, the strategic design of nanostructured catalysts is pivotal in boosting their stability. Specific formations, such as nanowires, nanorods, nanosheets, or more intricate porous nanomaterials, have proven effective in enhancing material resilience against deformation and chemical degradation under conditions of high industrial current densities [92]. For example, Guo et al. investigated an innovative method by combining NiFe-layered double hydroxide nanosheets with vertically aligned Mxene nanosheets [93], as illustrated in Figure 8c. This layered 3D electrode structure facilitated a significant enhancement in charge transfer rates through a pronounced synergistic effect among internal electrons. Moreover, this innovative electrode showcased exceptional durability, maintaining stable operation for 400 h.

Interestingly, combining 0D, 1D, and 2D nanostructures to form a 3D-layered self-supporting electrode emerges as an effective strategy. Yu and colleagues created a core-shell structure using copper nanowires, nickel-iron nanosheets, and Pt_3_Ir alloy nanoparticles [94], as shown in Figure 8d. Notably, this structure maintained its stability due to the forest-like arrangement of the copper nanowires, which withstood multiple treatments. The Pt_3_Ir nanoparticles remained evenly spread on the NiFe nanosheets. The resulting electrocatalyst showed a very low overpotential of 239 mV at 1000 mA cm^−2^ and demonstrated remarkable durability, with minimal potential changes after operating at 500 mA cm^−2^ for seven days (Figure 8e,f).

## 4. Conclusions and Outlooks

Electrochemical water splitting is considered a promising pathway to advance the field of hydrogen energy research. The fabrication of efficient and robust catalysts in industrially relevant environments is essential for water splitting. In this review, we focus on challenges and design strategies for high current density water splitting electrocatalysts. In terms of challenges, catalytic activity, mass diffusion and catalyst stability are the three main challenges for electrocatalysts. To address these challenges, a number of catalyst design strategies are proposed. First, the enhancement of the electrocatalyst active site activity and an increase in the number of electrocatalyst active sites lead to improved charge transfer capability. Second, the detachment of bubbles can be effectively promoted by designing superwetting structures, thereby improving mass diffusion at high current densities. Third, self-supporting electrodes were designed to improve the long-term stability of the electrocatalysts. The HER performance of the aforementioned electrocatalysts is summarized in Table 1. These electrocatalysts are also available for water-splitting electrolyzers and demonstrate excellent performance at current densities exceeding 500 mA cm^−2^ (Table 2).

**Table 1:** Summary of some nano electrocatalysts for high current density HERs.

Design Strategy	Electrocatalyst	Electrolyte	Activity (mV@mA cm^−2^)	Stability (h@mA cm^−2^)	Ref.
Electronic structure modulation	Ce-CoP/Ni_3_P	1 M KOH	225@1000	200@500	[61]
	Bi_2_O_3_-O_V_	1 M KOH	310@300	/	[64]
	HSD-PtNi/NF	1 M KOH	63@1000	300@1000	[65]
	MnCo/NiSe/NF	1 M KOH	211.6@1000	150@500	[70]
	Am-Mo-NiS_0.5_Se_0.5_	1 M PBS	209@1000	/	[71]
	MoO_2_@Ru	1 M KOH	131@1000	100@1000	[95]
	Pt/NiO_x_-O_V_	1 M KOH	≈180@500	100@1000	[96]
	Ru-NiSe_2_	1 M KOH	180.8@1000	90@1000	[97]
	Ru-Ni_3_N/NiO	1 M KOH	190@1000	1000@500	[98]
	Ru/Ni@C	1 M KOH	309@1000	100@1000	[99]
	Co/NC-HP@Si-NW	0.5 M H_2_SO_4_	440@500	24@500	[100]
	MoS_2_-P2	1 M KOH	332@500	240@500	[101]
Crystal phase modulation	MnO/CoP/NF	1 M KOH	259.5@1000	100@500	[102]
	Fe_2_P/Co_2_N	1 M KOH	131@500	40@500	[103]
	Mo_2_N/Ni_3_Mo_3_N	1 M KOH	123@500	120@500	[104]
	(Fe, Ni)_2_P@Ni_2_P	1 M KOH	255@1000	120@1000	[105]
	RuCo@Ru_SA_Co_SA_-NMC	1 M KOH	291@1500	576@1000	[106]
	PtNiMg	1 M KOH	/	100@2000	[107]
	Ni_2.86_Te_2_/NF	1 M KOH	348@200	/	[108]
	(WO_2_-Ni_17_W_3_)/ NiFe(OH)_X_/NF	1 M KOH	258@1000	120@1000	[109]
Superwetting structure	Ni(OH)x/Ni_3_S_2_	1 M KOH	238@1000	1000@320	[78]
	NCP/PC	1 M KOH	145@1000	1000@1000	[81]
	MoNi/NiMoO_x_	1 M KOH	139@1900	100@600	[110]
	HW-NiMoN/NF-2h	1 M KOH	107@1000	100@500	[111]
	Pt-Ni@NiMoN/NF	1 M KOH	90@500	120@1000	[112]
	Mo2N-Mo2C/N-CW	1 M KOH	311@500	/	[113]
Self-supported electrodes	Cu NWs@NiFe-Pt_3_Ir	1 M KOH	239@1000	168@500	[94]
	FeCoCrCuOx@CF	1 M KOH	/	160@500	[114]
	NMFSOH	1 M KOH	200@1000	300@500	[115]
	Fe_0.01_-Ni&Ni_0.2_Mo_0.8_N	1 M KOH	135@500	100@400	[116]
	NiFe-LDH@NiMo-H2@NF	1 M KOH	73@500	400@500	[117]
	IrNi-FeNi_3_/NF	1 M KOH	288.8@1000	124@1000	[118]
	Ni_5_P_4_-Co_2_P/NCF	1 M KOH	267@1000	100@250	[119]
	MnO_X_/NiFeP/NF	1 M KOH	255@500	120@500	[120]

**Table 2:** Summary of above nano electrocatalysts for water splitting electrolyzers (RT: room temperature).

Two-Electrode System	Condition	Activity (V@mA cm^−2^)	Stability (h@mA cm^−2^)
Ce_0.2_-CoP/Ni_3_P@NF||Ce_0.2_-FeP_X_/Ni_3_P	25 wt% KOH, 50 °C	1.718@500	200@500
HSD-PtNi/NF||NiFe LDH	1 M KOH, RT	1.72@500	/
MnCo/NiSe||MnCo/NiSe	1 M KOH, RT	1.88@1000	200@500
Am-Mo-NiS_0.5_Se_0.5_||Am-Mo-NiS_0.5_Se_0.5_	1 M PBS, RT	1.98@1000	/
MoO_2_@Ru NT||NiFe LDH	1 M KOH, RT	1.78@1000	200@1000
R-NF-Pt||NiFe-LDH	1 M KOH, RT	1.776@1000	400@1000
Ru-Ni_3_N/NiO||Ru-Ni_3_N/NiO	1 M KOH, RT	1.74@1000	1000@500
MnO-CoP/NF||MnO-CoP/NF	1 M KOH, RT	1.76@500	100@500
Fe_2_P/Co_2_N||Fe_2_P/Co_2_N	1 M KOH, RT	1.663@500	120@500
(Fe, Ni)_2_P@Ni_2_P||(Fe, Ni)_2_P@Ni_2_P	1 M KOH, RT	1.933@1000	12@1000
RuCo@RuSACoSA-NMC||RuO_2_	1 M KOH, RT	2.15@1000	7200@1000
Ni(OH)x/Ni_3_S_2_/NF||NiFe LDH/NF	1 M KOH, RT	1.83@1000	400@360
MoNi/NiMoO_X_||Co_2_(OH)_3_Cl/FeOOH	1 M KOH, RT	3.05@500	1600@200
FeCoCrCuO_X_/CF||FeCoCrCuO_X_/CF	1 M KOH, RT	2.95@500	100@500
Fe_0.01_-Ni&Ni_0.2_Mo_0.8_N||Fe_0.01_&Mo-NiO	6 M KOH, 60 °C	1.539@1000	80@425
NiFe-LDH@NiMo-H_2_@NF|| NiFe-LDH@NiMo-H_2_@NF	1 M KOH, RT	1.61@500	200@500
IrNi-FeNi_3_/NF||IrNi-FeNi_3_/NF	1 M KOH, 30 °C	1.78@500	100@500
MnO_X_/NiFeP/NF||MnO_X_/NiFeP/NF	1 M KOH, RT	1.828@1000	120@500

Despite significant progress in the development of efficient electrocatalysts for high-current-density water splitting, there is still a large gap between laboratory-scale studies and industrial-scale applications. Looking forward, many challenges are still urgent to be overcome.

(1) Scale-up production and synthesis of electrocatalysts: In high-current-density water splitting, although some excellent electrocatalysts exhibit good stability, they are complex to synthesize and difficult to scale up, limiting their use in commercial alkaline electrolyzers. These electrolyzers typically require large-area electrodes, which are difficult to meet with traditional laboratory-scale solvent heating and electrodeposition methods. To overcome these challenges, experimental designs need to consider the scalability of the synthesis methods. In addition, the development of new synthesis techniques, such as wet chemical methods and 3D printing, is also seen as a powerful way to achieve scale-up [121]. While pursuing technical feasibility, the economic and environmental impacts of the catalysts need to be considered to ensure the cost-effectiveness of the electrodes and the environmental sustainability of their production.
(2) Advances in in situ and operando characterization techniques: In electrocatalytic studies, phase characterization of catalysts is usually only possible in their stable final state [122], which limits our in-depth understanding of the micro-mechanisms of catalytic reactions. The development of in situ and operando characterization techniques is crucial for monitoring phase changes during catalysis, especially when it comes to unraveling the mechanism of HER. Although there are still current controversies regarding the mechanisms of HERs, in situ techniques can help resolve these controversies by providing direct evidence about the reaction intermediates.
(3) The environmental adaptability of electrocatalysts: The adaptation of electrocatalysts under different electrolyte conditions is one of the central issues of great interest in the field of electrochemistry today. Although most studies have focused on their performance under alkaline conditions, their behavior under acidic and neutral environments has not been fully appreciated [123]. Understanding the differences in the performance of electrocatalysts under different pH environments is crucial, not only for hydrogen energy technology and water utilization but also for the study of other electrocatalytic reactions. In order to achieve this goal, extensive performance evaluations are needed, including electrocatalytic activity in acidic, neutral, and alkaline electrolytes, as well as stability and efficiency tests under simulated industrial conditions. In addition, standardized catalyst test methods and seawater electrolysis components need to be developed in order to advance electrocatalyst technology toward practical applications. This will help ensure consistent and comparable evaluations and provide a solid foundation for future electrocatalyst design and optimization.
(4) Innovations in electrode design: The key to innovation in electrode design is to optimize the hydrophilic and hydrophobic properties of the electrode and introduce novel structures, such as 3D self-supporting electrodes, to enhance electrolysis efficiency and long-term stability [124]. By adjusting the chemical composition and microstructure of the electrode surface, such as micro- or nano-scale roughness, we can significantly improve the hydrophilicity of the electrode, which helps to increase the contact with the aqueous electrolyte and facilitates ion transport. Meanwhile, the superhydrophobic design, such as through nano-arrays and layered structures, can reduce the adhesion of gas bubbles on the electrode surface and accelerate the gas emission, thus improving the gas transfer efficiency of the overall electrolysis process. In addition, optimal activity and stability can be achieved by optimizing the structure and composition of the electrocatalysts at the atomic, nano, and micro scales. These multiscale design strategies not only improve the performance of electrodes but also help to improve the matching of electrolyzer and electrodes and promote the widespread adoption of electrolysis technology in industrial applications.
(5) Systematic study of temperature and pressure: An in-depth study of catalyst performance under different environmental conditions, especially taking into account temperature and pressure variations [125], is essential for optimizing electrocatalytic processes in industrial applications. In practice, catalysts undergo significant changes in active sites and structure under the influence of high temperatures and pressures, and these changes can significantly affect electrocatalytic performance and reaction kinetics [126]. Therefore, by simulating these harsh industrial conditions and evaluating the catalysts, it can help us to gain a deeper understanding and improve the design and functionality of electrocatalysts to meet industrial standards and efficiency requirements.
